# Spectrum of Oncogenic Driver Mutations in Lung Adenocarcinomas from East Asian Never Smokers

**DOI:** 10.1371/journal.pone.0028204

**Published:** 2011-11-30

**Authors:** Chenguang Li, Rong Fang, Yihua Sun, Xiangkun Han, Fei Li, Bin Gao, A. John Iafrate, Xin-Yuan Liu, William Pao, Haiquan Chen, Hongbin Ji

**Affiliations:** 1 State Key Laboratory of Cell Biology, Institute of Biochemistry and Cell Biology, Shanghai Institutes for Biological Sciences, Chinese Academy of Sciences, Shanghai, China; 2 Department of Thoracic Surgery, Fudan University Shanghai Cancer Center, Shanghai, China; 3 Department of Oncology, Shanghai Medical College, Shanghai, China; 4 Department of Pathology, Cancer Center, Massachusetts General Hospital, Boston, Massachusetts, United States of America; 5 Vanderbilt-Ingram Cancer Center, Nashville, Tennessee, United States of America; Maastricht University Medical Center, The Netherlands

## Abstract

**Purpose:**

We previously showed that 90% (47 of 52; 95% CI, 0.79 to 0.96) of lung adenocarcinomas from East Asian never-smokers harbored well-known oncogenic mutations in just four genes: *EGFR*, *HER2*, *ALK*, and *KRAS*. Here, we sought to extend these findings to more samples and identify driver alterations in tumors negative for these mutations.

**Experimental Design:**

We have collected and analyzed 202 resected lung adenocarcinomas from never smokers seen at Fudan University Shanghai Cancer Center. Since mutations were mutually exclusive in the first 52 examined, we determined the status of *EGFR, KRAS, HER2, ALK,* and *BRAF* in stepwise fashion as previously described. Samples negative for mutations in these 5 genes were subsequently examined for known *ROS1* fusions by RT-PCR and direct sequencing**.**

**Results:**

152 tumors (75.3%) harbored *EGFR* mutations, 12 (6%) had *HER2* mutations, 10 (5%) had *ALK* fusions all involving *EML4* as the 5′ partner, 4 (2%) had *KRAS* mutations, and 2 (1%) harbored *ROS1* fusions. No *BRAF* mutation were detected.

**Conclusion:**

The vast majority (176 of 202; 87.1%, 95% CI: 0.82 to 0.91) of lung adenocarcinomas from never smokers harbor mutant kinases sensitive to available TKIs. Interestingly, patients with *EGFR* mutant patients tend to be older than those without *EGFR* mutations (58.3 Vs 54.3, *P* = 0.016) and patient without any known oncogenic driver tend to be diagnosed at a younger age (52.3 Vs 57.9, *P* = 0.013). Collectively, these data indicate that the majority of never smokers with lung adenocarcinoma could benefit from treatment with a specific tyrosine kinase inhibitor.

## Introduction

Lung cancer is the leading cause of cancer-related death worldwide [1], [2]. Although the majority of cases occur in those with a personal history of tobacco smoke exposure, lung cancer also occurs in never smokers, defined as individuals who smoked less than 100 cigarettes in a lifetime. If lung cancer from never smokers was considered as a separate category, the disease would rank among seven to nine most common fatal cancers in the US[3]. While it was reported that up to 30% of lung cancer patients in East Asia were never smokers, it would rank the fifth most common malignancies in China as a separate disease[4], [5].

Lung cancer in never smokers is clinically distinct from other subsets of the disease. First, although lung cancer is comprised of four main histologies including small cell carcinoma, squamous cell carcinoma, large cell carcinoma, and adenocarcinoma, about 70% of never smokers have adenocarcinoma[6]. Second, women have higher risk of developing lung cancer than men among never smokers[3], [7]. Third, never smokers comprise a higher percentage (∼30%) of patients with lung cancer in East Asian countries versus North American and European populations (∼10%)[4], [6]. Reasons for this discrepancy are unknown.

At the molecular level, lung cancer in never smokers is also unique. From genetic analysis of 52 adenocarcinomas samples from patients who never smoked, we previously showed that approximately 90% of samples harbor mutually exclusive driver alterations in just four genes: *EGFR*, *KRAS*, *HER2*, and *ALK* [8]. By contrast, only ∼40% of cases of non-small cell lung cancer (NSCLC, which includes squamous cell carcinoma, large cell carcinoma, and adenocarcinoma histology) would harbor such mutations.

In this new study, we have further collected another 150 never smoker tumor samples besides the 52 samples previously published for detection of known driver mutations including *EGFR, KRAS, HER2, BRAF,* and *EML4*-*ALK* alterations [8]. Furthermore, we screened ‘pan-negative’ samples for the presence of *ROS1* fusions. *ROS1* is the human homolog of the avian sarcoma virus UR2 transforming gene v-ros and encodes a receptor tyrosine kinase (RTK) of the insulin receptor family. Activating *ROS1* fusions (involving the *FIG* gene) were previously found in glioblastoma [9] and more recently in lung cancer [10]. We chose to focus first on *ROS1* because cell lines harboring *ROS1* fusions are sensitive to the tyrosine kinase inhibitor, crizotinib (Bergethon, Pao, Ji, Chen, Iafrate, et al, submitted), making it another potentially targetable mutant kinase in the disease. This study will hopefully provide important insights into molecular defects and identify therapeutic targets in never smoker patients with lung adenocarcinomas.

## Materials and Methods

### Patients and tissues

Primary tumor samples were obtained from 1103 consecutive patients who underwent potentially curative pulmonary resection at the Fudan University Shanghai Cancer Centre from Oct 2007 through Sep 2010. This study was approved by the Institutional Review Board of the Fudan University Shanghai Cancer Center, Shanghai, China. All participants gave written informed consent. We further collected 150 never smoker tumor samples besides the 52 samples that have been published [8]. A total of 202 patients were enrolled in this specific study based upon the following criteria: they are all never smokers (defined as smoked less than 100 cigarettes in their lifetime), they had a pathologic diagnosis of lung adenocarcinoma, their tumor sample contained a minimum of 50% tumor cells as determined by study pathologists, they did not receive neoadjuvant chemotherapy, and they had sufficient tissue for molecular analysis.

### RNA extraction and mutational analysis

All mutational analyses were performed in China. Frozen tissues were grossly dissected into TRIZOL (Invitrogen Inc.) for RNA extraction following standard protocols. Total RNA samples were reverse transcribed into single-stranded cDNA using RevertAid™ First Strand cDNA Synthesis Kit (Fermentas, EU).

*EGFR* (exons 18–22), *HER2* (exons 18 to 21), *KRAS* (exons 2 to 3), and *BRAF* (exons 11 to 15) were PCR amplified using cDNA and directly sequenced. For detection of *EML4-ALK* fusions, primers were designed to amplify all known fusion variants using cDNA. The forward primers were *EML4* E2F (5′-TGATGTTTTGAGGCGTCTTG-3′), *EML4* E13F (5′-AGATCGCCTGTCAGCTCTTG-3′), and *EML4* E18F (5′-TTAGCATTCTTGGGGAATGG-3′), and the reverse primer *ALK* E20R was (5′-TGCCAGCAAAGCAGTAGTTG-3′). Primers used to detect fusions between *ALK* and *KIF5B* or *TFG* were as previously reported [11], [12]. For detection of *CD74*-*ROS1* and *SLC34A2-ROS1* fusions, the forward primers were *CD74* E5F (5′-CCTGAGACACCTTAAGAACACCA-3′) and *SLC34A2* E4F (5′-TCGGATTTCTCTACTTTTTCGTG-3′). The reverse *ROS1* primer was E34R (5′-TGAAACTTGTTTCTGGTATCCAA-3′).

Multiplex PCR analysis was done with KOD plus DNA polymerase (Toyobo, Osaka, Japan). The program to detect *ALK* fusions was : 94°C 5 minutes; 94°C 30 seconds, 63°C 30 seconds, 68°C 1 minute, 35 cycles; 68°C 10 minutes. The program to detect *ROS1* fusions was: 94°C 5 minutes; 98°C 10 seconds, 62°C 30 seconds, 68°C 15 seconds, 35 cycles; 68°C 10 minutes. PCR products were directly sequenced in both forward and reverse directions. All mutations were verified by analysis of an independent PCR isolate.

### Real-time PCR quantification

The level of *ROS1* mRNA was determined using Platinum® SYBR® Green qPCR SuperMix-UDG (Invitrogen, CA, USA). The primers for real-time PCR were *ROS1*-qPCR-F (5′-CAAGAACCCGACCAAAGACCTAC-3′) and *ROS1*-qPCR-R (5′-CAAATCACATCGCCATCTTCACC-3′). The results were analyzed and expressed as relative mRNA expression of CT (Threshold Cycle) value, which was then converted to fold changes.

### Statistical analysis

Associations between mutations and clinical and biological characteristics were analyzed by *χ^2^* or Fisher's exact test. All data were analyzed using the Statistical Package for the Social Sciences Version 16.0 Software (SPSS Inc., Chicago, IL). The two-sided significance level was set at *p*<0.05.

## Results

### Patient Characteristics

From Oct 2007 to Sep 2010, we consecutively collected a total of 452 resected lung adenocarcinomas. All patients were Chinese. Interestingly, there were 285 from never smokers while only 167 were from smokers,highlighting a predominant percentage (63.1%) of never smokers in this specific subtype of lung cancer. 202 lung adenocarcinomas from never smokers met eligibility for this study. The median age at diagnosis was 57.3 years **(** **Table 1** **)**. The number of patients in stages I-IV was 113, 21, 62 and 6, respectively. 43 tumors were from males and 159 from females. No differences were observed in age, stage, or degree of tumor differentiation between males and females.

**Table 1:** Clinical Characteristics of Never Smokers With Lung Adenocarcinomas.

		Sex		
Characteristics	Total	Male	Female	*P*
No. of patients	202	43	159	
Age, years	57.3	59.9	56.6	.061
SD	10.1	11	9.8	
Clinical Stage				
I	113	18	95	.058
II	21	7	14	
III	62	15	47	
IV	6	3	3	
Differentiation				
Well	38	8	30	.160
Moderate	111	19	92	
Poor	53	16	37	

### *EGFR* mutation status

**Figure 1:**
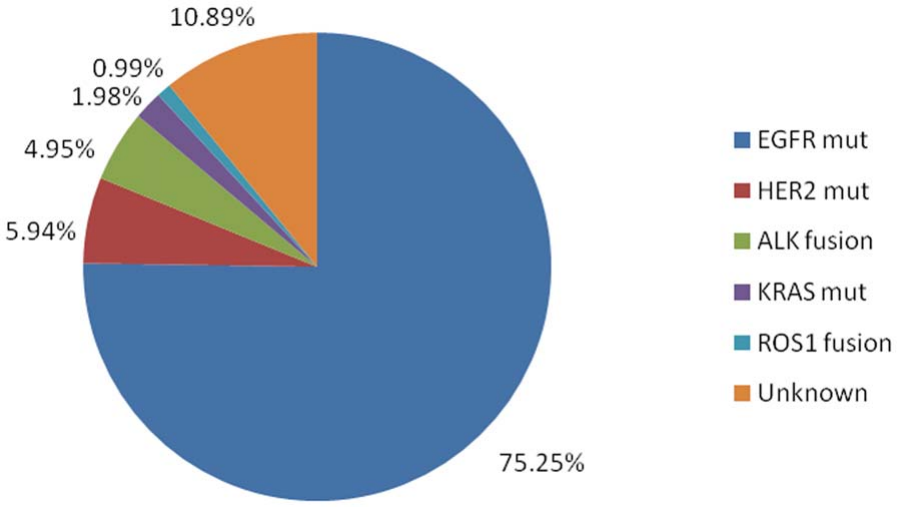
Spectrum of oncogenic driver mutations in lung adenocarcinomas from never smokers. From 202 tumors, 75.3% (152/202) harbored *EGFR* kinase domain mutations, 5.9% (12/202) *HER2* mutations, 5.0% (10/202) *ALK* fusions, and 2% (4/202) *KRAS* mutations, 1% (2/202) of tumors harbor *ROS1* fusion. There are 10.9% (22/202) with unknown oncogenic driver mutations.

75.3% (152/202) of tumors were found to harbor *EGFR* kinase domain mutations **(** **Figure 1** **)**. Among these, 77 were deletions in exon 19 and 59 were L858R missense changes. Other alterations included 7 exon 20 insertions and 4 exon 18 G719X mutations. 2 samples from patients without previous chemotherapy or TKI treatment harbored concurrent L858R and T790M mutations. Other *EGFR* mutations included L816Q, I768S, E709K, and K757M.

Tumors from 76.7% (122/159) of female never smokers harbored *EGFR* kinase domain mutations. A comparable *EGFR* mutation rate (69.8%, 30/43) was found in male never smokers (*P* = 0.348). This confirmed our previous study [13] showing no significant difference in the frequency of *EGFR* mutations between men and women with lung adenocarcinoma who never smoked. The average age at diagnosis between patients with *EGFR* mutant and wild-type tumors was 58.3 and 54.3 years, respectively (*P* = 0.016), showing that patients with *EGFR* mutant patients tend to be older than those without *EGFR* mutations. The *EGFR* mutation rate in well, moderate and poorly differentiated tumors was 86.8%, 75.7% and 66% respectively (*P* = 0.075) **(** **Table 2** **)**. There were no statistically significant differences among subtypes of *EGFR* mutation and the analyzed clinicopathological features **(** **Table 3** **)**.

**Table 2:** Association between *EGFR* tyrosine kinase mutations and clinical pathological features.

Characteristics	Total	Mutant	Wild type	*P*
Age, years	57.3	58.3	54.3	.016
SD	10.1	9.8	10.4	
Gender				
Male	43	30	13	.348
Female	159	122	37	
Clinical Stage				
I	113	87	26	.831
II	21	16	5	
III	62	44	18	
IV	6	5	1	
Differentiation				
Well	38	33	5	.075
Moderate	111	84	27	
Poor	53	35	18	

**Table 3:** Correlation Between *EGFR* Mutation Subtypes and Clinical Pathological Features.

		Subtypes of *EGFR* mutation			
Characteristics	Total	Exon 19 deletion	L858R	Others	*P*
Age, years	58.3	57.1	60.6	55.4	0.056
SD	9.8	10.2	8.9	10.4	
Gender					
Male		13	13	4	0.608
Female		64	46	12	
Clinical Stage					
I		42	36	9	0.899
II		10	5	1	
III		23	16	5	
IV		2	2	1	
Differentiation					
Well		11	15	7	0.073
Moderate		46	30	8	
Poor		20	14	1	

### *HER2*, *KRAS*, *BRAF*, and *ALK* alterations

5.9% (12/202) of samples had *HER2* kinase domain mutations, among which 11 had exon 20 insertions and 1 had an L755P point mutation. *EML4-ALK* fusions were found in 5% (10/202) of samples, involving EML4 exons 13, 20, and 6 (V1, V2 and V3a/b variants). No *KIF5B*-*ALK* or *TFG*-*ALK* fusions were detected. Four samples (2%) had a *KRAS* mutation, including two G12V and two G12D mutations, respectively. No mutations were found in *BRAF* **(** **Figure 1** **).** Clinical characteristics associated with these different oncogenic driver mutations are shown in **Table 4**.

**Table 4:** Association Between Driver Mutations and Age and Gender in Lung Adenocarcinoma From Never Smokers.

	*HER2*			*EML4*-*ALK*			*KRAS*			*ROS1*		
Characteristics	Mutant	Wild type	*P*	Mutant	Wild type	*P*	Mutant	Wild type	*P*	Mutant	Wild type	*P*
Age, years	52.8	57.6	0.107	59.3	57.3	0.540	62.0	57.3	0.359	46.5	57.5	0.128
SD	5.4	10.3		9.8	10.2		3.4	10.2		2.1	10.2	
Gender												
Male	1	42	0.467	4	39	0.228	3	40	0.032	0	43	1.000
Female	11	148		6	150		1	155		2	154	

### *ROS1* fusions

**Figure 2:**
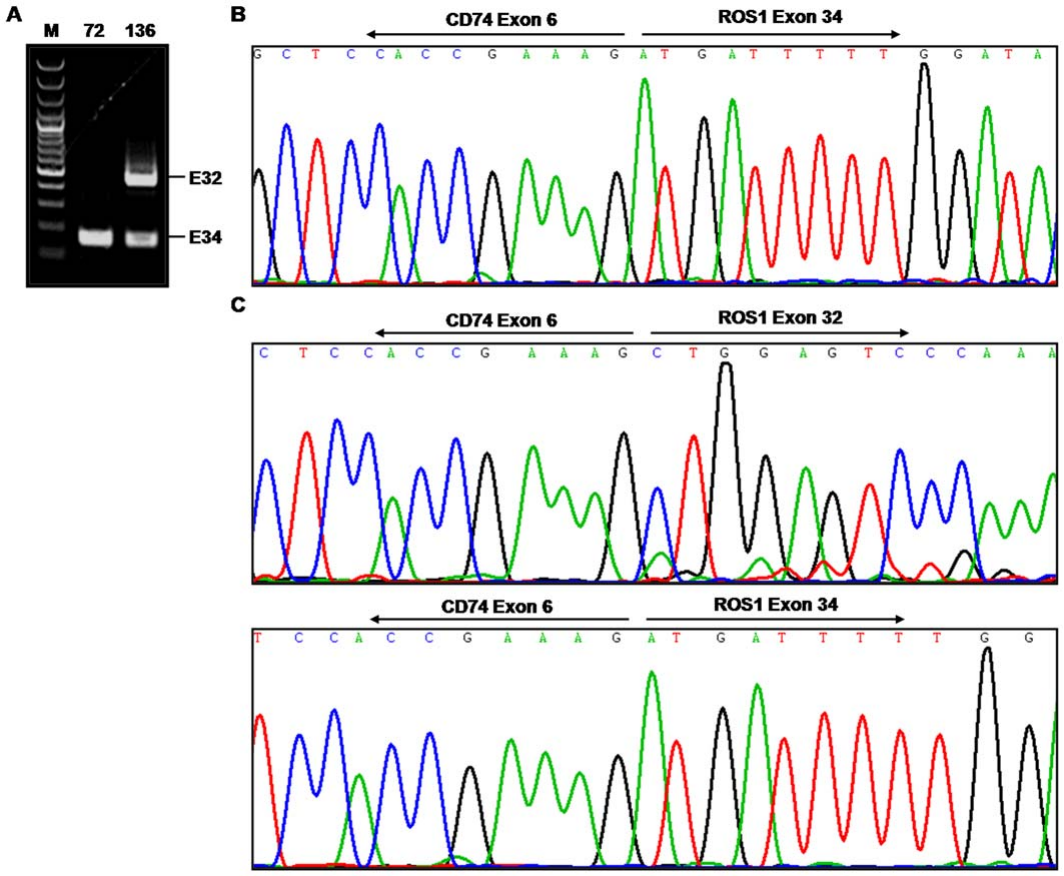
Detection of *CD74-ROS1* fusions in lung adenocarcinomas from never smokers. (A) Agarose gel electrophoresis analysis of RT-PCR products for *CD74*-*ROS1* fusions. E32, CD74-ROS1 exon 32 fusion; E34, CD74-ROS1 exon 34 fusion. (B) Sequencing of RT-PCR product from a tumor (No.72) identified a fusion of *CD74* exon 6 to *ROS1* exon 34. (C) Sequencing of RT-PCR product from a tumor (No.136) identified a fusion of *CD74* exon 6 to both *ROS1* exon 32 and exon 34.

**Figure 3:**
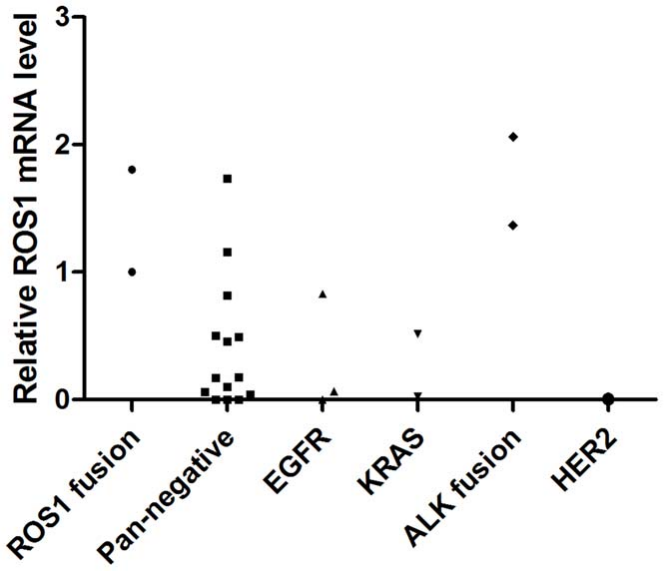
Detection of ROS1 mRNA levels in lung adenocarcinomas from never smokers either with indicated oncogenic driver mutations or negative for all known oncogenic driver mutations.

1% (2/202) of samples had *CD74-ROS1* fusions **(** **Figure 2A** **)**. No fusions involving *SLC34A2* were detected. Both patients with *ROS1* rearrangements were female and diagnosed with stage III disease, and both harbored the previously described *CD74-ROS1* fusion involving exon 34 of *ROS1* fused to exon 6 of *CD74* **(** **Figure 2B** **)**. Interestingly, one of the samples (No.136) had two types of *CD74-ROS1* fusions: exon 32 or exon 34 of *ROS1* fused to exon 6 of *CD74* (Fig. 2C). Both fusions were in-frame and retained the transmembrane region of *ROS1*, which may be different splicing products produced from the same translocation event similar to *SLC34A2-ROS1* fusions previously found in HCC78 cells [10]. To test if the high *ROS1* expression is associated with the *ROS1* fusion, we have performed the Q-PCR experiment in 24 samples including the 2 *ROS1*-fusion positive samples, 3 *EGFR* mutated samples, 2 *KRAS* mutated samples, 2 *ALK* fusion positive samples, 1 *HER2* mutated sample and 14 pan-negative samples defined as without above driver mutations. Interestingly, both *ROS1*-fusion positive samples did show relative high *ROS1* expression **(** **Figure 3** **)**. However, other samples with either *ALK* fusion or *EGFR* mutations or without any known oncogenic drivers also showed comparable expression of *ROS1* **(** **Figure 3** **)**, indicating that the *ROS1* mRNA level may not be as an good indicator for the *ROS1* fusion.

### Pan-negative samples

Despite identification of *ROS1* rearrangements, still 10.9% (22 of 202) samples had no identifiable driver mutation **(** **Figure 1** **)**. These data are consistent with our previously published study[8]. Interestingly, further comparative analyses showed that patients without oncogenic driver mutation tend to be diagnosed at a younger age than those with known oncogenic drivers (52.27 Vs 57.93, *P* = 0.013) **(** **Table 5** **).**

**Table 5:** Comparison of clinical Characteristics of patients with or without known driver mutations.

	With known driver mutations	Without known driver mutations	*P* value
Age, years	57.9	52.3	0.013
SD	9.6	12.7	
Gender			
Male	38	5	0.789
Female	142	17	
Clinical Stage			
I–II	118	16	0.502
III–IV	62	6	
Differentiation			
Well	36	2	0.441
Moderate	98	13	
Poor	46	7	

## Discussion

During the past decade, a wealth of data from genomic[14], expression[15], mutational[16], and proteomic profiling studies[10] have led to the identification of multiple molecularly distinct subsets of non-small cell lung cancer (NSCLC). Based upon these findings, one of the most promising treatment strategies now involves the subdivision of NSCLC histologies into clinically relevant molecular subsets, using a classification schema based upon specific ‘driver mutations’. Major recurrent mutations in lung adenocarcinoma have been found to occur in *EGFR*, *KRAS*, *HER2*, *BRAF*, *ALK* and *ROS1*.

In our previous study, approximately 90% of lung adenocarcinomas from never smokers harbor known oncogenic mutations in just 4 genes of *EGFR*, *KRAS*, *HER2*, *EML4*-*ALK* [8]. Here we comprehensively analyzed all the known oncogenic drivers up to date including all known types of *ALK* fusions besides *EML4*-*ALK* and *ROS1* fusions and expanded the sample size. We show that 89.1% of samples harbor known oncogenic driver mutations, consistent with our previous report[8]. In the present study, *EGFR* kinase domain mutations were found in 75.3% of samples. *EGFR* kinase domain mutations are more common in elder patients (*P* = 0.016), consistent with previous studies[17].

The mutation spectrum including *EGFR*, *HER2*, *KRAS*, *EML4*-*ALK* in this study shows similar proportions as previously described[8]. In an attempt to provide a more comprehensive map of oncogenic drivers, we have examined other types of *ALK* fusions, such as *KIF5B*-*ALK* and *TFG*-*ALK*. However, no tumor positive for these two *ALK* fusions was found. We have identified 12 samples with *HER2* kinase domain mutations, including 11 exon 20 insertions and 1 L755P point mutation. A previous study showed that lung adenocarcinoma patients harboring *HER2* exon 20 insertion responded dramatically to Trastuzumab, a monoclonal antibody to *HER2* [18]. Pan-ErbB family inhibitors such as Neratinib and BIBW2992[19], [20], under clinical trials to overcome *EGFR* T790M mutation, may also be used in patients harboring *HER2* mutations. Except for *KRAS,* all the other oncogenic driver mutations have targeted agents being used in clinic or under clinical trials.

*ROS1* fusion is recognized as a new oncogenic driver [10]. Here we found about 1% of samples harbored *CD74*-*ROS1* fusion. Interestingly, there is one sample with two forms of *ROS1* fusion, which may be derived from the same rearrangement due to alternative splicing mechanism as previously reported[10]. However, it remains unknown why one tumor harbors two forms of fusion and if there is any synergy between them. *ROS1* fusion has been shown to contribute to the formation of lung adenocarcinoma [10]. Knockdown of *ROS1* in the lung cancer cell line HCC78 which has *SLC34A2*-*ROS1* fusion promotes apoptosis [10], indicating *ROS1* fusion is important for cell survival. This validates *ROS1* as a good target for clinical treatment. Lung cancer cell line HCC78 positive for *ROS1* fusion is sensitive to the tyrosine kinase inhibitor, crizotinib (Bergethon, Pao, Ji, Chen, Iafrate, et al, submitted), indicating that ROS1 fusion is another potentially targetable mutant kinase for lung cancer.

In summary, we have expanded and extended our previous study and examined all known oncogenic driver mutations up to date in lung adenocarcinomas from a large cohort of never smokers. Most of the patients could be classified to a certain type according to oncogenic driver mutations. Except for KRAS, all oncogenic drivers can be effectively targeted in clinic, highlighting the importance of molecular classification of lung adenocarcinoma in never smokers. Interestingly, patients without any oncogenic driver mutation tend to be diagnosed at a younger age. Although we do not have an explanation yet, we are now studying these ‘Pan-negative’ samples using whole exome sequencing in a hope to identify certain novel oncogenic drivers. Our study suggest that most lung adenocarcinoma patients who never smoked could potentially benefit from personalized targeted therapy.
